# SAbDab: the structural antibody database

**DOI:** 10.1093/nar/gkt1043

**Published:** 2013-11-08

**Authors:** James Dunbar, Konrad Krawczyk, Jinwoo Leem, Terry Baker, Angelika Fuchs, Guy Georges, Jiye Shi, Charlotte M. Deane

**Affiliations:** ^1^Department of Statistics, University of Oxford, 1 South Parks Road, Oxford OX1 3TG, UK, ^2^Informatics, UCB Pharma, 216 Bath Road, Slough SL1 4EN, UK and ^3^Roche Pharma Research & Early Development, Roche Diagnostics GmbH, 82377 Penzberg, Germany

## Abstract

Structural antibody database (SAbDab; http://opig.stats.ox.ac.uk/webapps/sabdab) is an online resource containing all the publicly available antibody structures annotated and presented in a consistent fashion. The data are annotated with several properties including experimental information, gene details, correct heavy and light chain pairings, antigen details and, where available, antibody–antigen binding affinity. The user can select structures*,* according to these attributes as well as structural properties such as complementarity determining region loop conformation and variable domain orientation. Individual structures, datasets and the complete database can be downloaded.

## INTRODUCTION

Antibodies form the foundations of the vertebrate immune response. These proteins form complexes with potentially pathogenic molecules called antigens and inhibit their function or recruit other components of the immunological machinery to destroy them. In addition to the biological importance of antibodies, their ability to be raised against an almost limitless number of molecules has made them useful laboratory tools and increasingly useful as therapeutic agents in humans (1). This biopharmaceutical application has motivated the desire to understand how binding, stability and immunogenic properties of the antibody are determined and how they can be modified.

Computational analyses and tools are increasingly being employed to aid the antibody engineering process (2). Many of these tools now use only the antibody data, as opposed to general protein data, because this has been shown to increase performance (3,4).

The publicly available structural data for most types of proteins are too sparse to merit protein-specific prediction methods. However, since the first antibody structure was deposited in 1976 (5), the number of antibody structures in the protein data bank (PDB) (6) has grown, and it now represents approximately 1.75% of the total 91939 entries (July 2013).

Several databases that handle antibody data currently exist (7–13). Of these, most are sequence-based or are antibody discovery tools. The most recent, DIGIT (13), provides sequence information for immunoglobulins and has the advantage over earlier sequence databases [Kabat (7), IMGT (9), Vbase2 (8)] of providing heavy and light chain sequence pairings. However, it does not incorporate structural data. AntigenDB (11) and IEDB-3D (12) do include structural data. However, both focus on collecting epitope data and do not include unbound antibody structures. In comparison, both IMGT (9) and the Abysis portal (10) provide the ability to inspect and download individual bound and unbound antibody structures. Neither allow for the generation of bespoke datasets nor for the download of an ensemble of curated structural data.

To address this problem, we have developed a Structural Antibody Database (SAbDab), a database devoted to automatically collecting, curating and presenting antibody structural data in a consistent manner for both bulk analysis and individual inspection. SAbDab updates on a weekly basis and provides users with a range of methods to select sets of structures. For example, users can select by species, experimental details (e.g. method, resolution and r-factor), similarity to a given antibody sequence, amino-acid composition at certain positions and antibody–antigen affinity. Entries can also be selected using structural annotations including, for example, the canonical form of the complementarity determining regions (CDR) (14), orientation between the antibody variable domains (15) and the presence of constant domains in the structure. Structures can be inspected individually or downloaded *en masse* either as the original file from the PDB or as a structure that has been annotated using the Chothia numbering scheme (16). In all cases, a tab-separated file detailing heavy and light chain pairing, antibody–antigen pairing and all other annotations is generated.

### Antibody structure nomenclature

**Figure 1.**
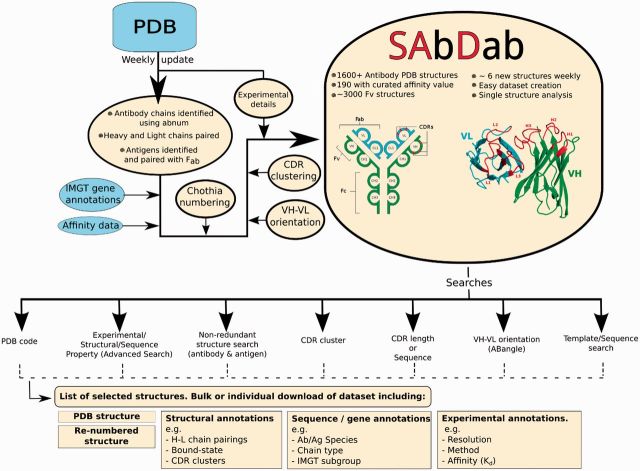
SAbDab’s workflow. Each week new structures from the PDB are analyzed to find antibody chains. These structures are then annotated with a number of properties and stored in SAbDab. Users may access and select this data using a number of different criteria. Structures and annotations can be downloaded individually or as a dataset. Inset, a schematic of the IgG antibody structure and the *F_v_* fragment formed by the heavy and light variable domains, VH and VL.

Antibodies have a well-defined structure that is conserved over majority of the molecule. They typically consist of four polypeptide chains, two light chains and two longer heavy chains (see Figure 1). Each light chain folds to form two domains, one variable (VL) and one constant (CL). Each heavy chain folds to form four or more domains, one variable (VH) and three or more constant domains (CH1, CH2 and CH3). The VL and CL1 domains from one light chain associate with the VH and CH1 domains of a heavy chain to form an antigen-binding fragment (*F_AB_*). Two *F_AB_*s form the arms of the Y-shaped structure of the antibody. The remaining constant domains on each heavy chain (CH2 and CH3) associate to form the stem of the Y and are known collectively as the crystallisable or constant (*F_C_*) fragment.

Typically, a natural antibody has two identical antigen-binding sites, one at the tip of each *F_AB_* arm. On both domains, VH and VL, of the variable fragment (collectively termed the *F_V_*) are three CDRs: H1, H2 and H3 on VH and L1, L2 and L3 on VL. Five of the six CDRs have structures that can be classified into ‘canonical clusters’ (16). The remaining loop, H3, is more variable and cannot be treated in the same way (17). In fact, modelling the H3 loop remains one of the most difficult challenges in antibody structure prediction (2).

The residues in each variable domain outside the CDRs are referred to as the framework regions. The framework is relatively conserved in sequence and has a β-sandwich architecture. This conserved structure allows for equivalent residue positions to be annotated from antibody to antibody. Several numbering systems exist that are largely similar over the framework region but have different definitions around the CDRs. Here, we primarily use the widely adopted Chothia numbering scheme (16), as it is informed by structural analysis and is defined over the entire variable region.

## DATA SOURCES AND CONTENTS

### Antibody structures

As of 25 July 2013, the database contains 1624 structures with one or more antibody chains. Of these, 1418 have at least one paired heavy and light chain that form a *F_AB_*. The remainder are largely single-domain antibodies or are cases when only one antibody chain has been crystallized. SAbDab is updated on a weekly basis using the technique summarized in Figure 1 and detailed below. The database is currently growing at a mean rate of six new structures per week.

Each week, the PDB releases new experimental structures. Using key word searches, it is possible to identify most of those that contain an antibody chain. However, no direct or consistent information is given about chain type, heavy–light chain pairings or antibody–antigen chain pairings. Therefore, SAbDab attempts to apply the Chothia antibody numbering to the sequence of each new chain using ABnum (18). This automatically detects each chain’s type—heavy, light or non-antibody. The process is applied recursively to sequences to identify each variable region of the chain and thus enable the identification of single-chain *F_v_*s (scFvs) that have not been split into separate chains. Those non-antibody chains that belong to a PDB entry containing an unequal number of heavy and light chains are aligned to antibody sequence profiles using MUSCLE (19). A chain must have a sequence identity of <35% to any antibody sequence profile for it to be considered a potential antigen. Those that exceed this threshold are flagged for manual inspection. In addition, any structure whose header details contain words similar to ‘T-cell’ or ‘MHC’ are flagged for manual inspection before their inclusion in SAbDab.

To pair heavy and light chains, the constraint is applied that the conserved cysteine at Chothia position 92 on a heavy chain must be within 22 Å of the conserved cysteine at position 88 on a light chain. Potential antigens are identified from the non-antibody chains and the non-polymer, nucleic-acid or carbohydrate molecules. Those small molecules that are recognized as common solvents (20) (e.g. glycerol) are discarded. Antibody chains are then paired with their antigen molecules by calculating the number of CDR residues that are within 7.5 Å of each candidate. If there is more than one molecule that makes contact with the antibody CDRs, the structure is flagged for manual inspection. Polypeptide antigens are classified as proteins if they contain >50 amino acids and peptides otherwise. Only the bound polypeptide chains are reported as the antigen. Other antigens are either classified as carbohydrates, nucleic acids or haptens (non-polymeric ligands). The antibody–antigen complex content (July 2013) is summarized in Table 1.

**Table 1.** SAbDab’s current antibody–antigen complex content

Antigen type	No. of structures	No. of *F_V_* regions
Protein	604	1081
Peptide	224	321
Carbohydrate	64	86
Nucleic-acid	12	15
Hapten	147	224
Total contents	1624	3048

Multiple *F_V_* regions may appear in a single PDB structure.

Annotations are obtained for the antibodies and antigens from a number of external sources. If the entry exists in the IMGT database, annotations of allele, gene, subgroup, group and isotype are collected. Where no IMGT entry exists, each antibody chain is annotated down to the subgroup level by alignment to representative sequences. Experimental details are collected from the PDB. Details about the name, molecule type and structure of non-peptide ligands are obtained from the ligand-expo database (6).

### Affinity data

Antibody binding affinity data are primarily obtained from two databases, PDB-Bind (21) and the structure-based benchmark (22). All the antibody entries were selected and only those with *K_A_* or *K_D_* data were kept. Where available, meta-data that are pertinent to affinity data (e.g. experimental conditions) are also collected.

Currently, SAbDab contains 190 structures with an associated affinity value. In total, 133 are bound to proteins, 38 to peptides and 19 to hapten antigens. This curated data set should serve as a useful benchmarking resource for the antibody–antigen docking prediction community and the antibody engineering community.

### Complementarity determining regions

There are multiple characterizations of antibody CDRs (16,23–25). In SAbDab, the Kabat (23), Contact (24) and Chothia (16) CDRs are annotated. The length and sequence of the CDRs, according to these three definitions, is extracted for each structure and recorded in SAbDab. In the database, the Chothia CDRs (16) are further analyzed to assign membership into structural clusters, often referred to as canonical conformations.

**Figure 2.**
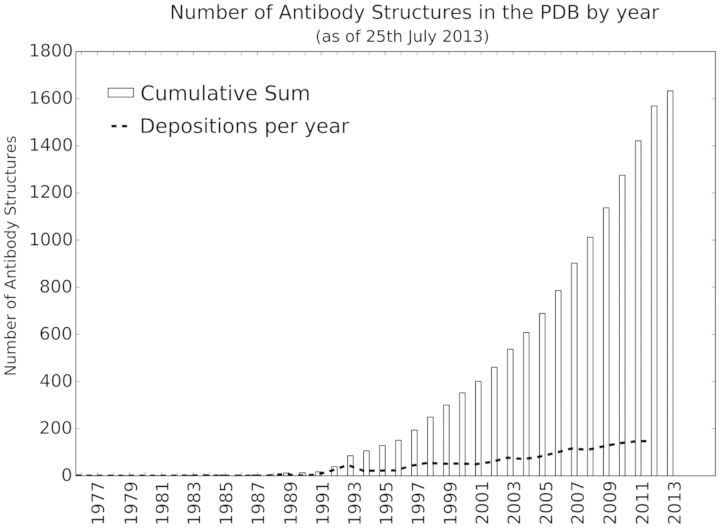
The number of antibody structures in the PDB is rising rapidly. On an average, six new antibody structures are added to SAbDab each week.

The canonical conformations of a given CDR type and length were originally created with the aim of linking sequence with structure. These groupings have been studied extensively (14,16,26–29). Given the exponential growth of the number of antibody structures in the PDB (Figure 2), we provide a standardized tool for studying the structural classes of CDRs. SAbDab regularly clusters the latest set of Chothia CDRs for each type (H1, H2, H3, L1, L2 and L3) and length. The clustering is performed by calculating the pairwise root mean square deviation between the CDRs and using a UPGMA clustering algorithm (30) at a number of cut-offs. Any correspondence to previously defined canonical classes is noted for each cluster. This feature will automatically monitor the conformational space of the CDRs as the amount of antibody structural data continues to increase.

### VH–VL orientation

The antigen binding site is formed between the variable domains, VH and VL, of an antibody. The topology of the site is therefore influenced by how the domains are orientated with respect to one another. Optimizing the VH–VL orientation has been proposed as a mechanism to fine tune antibody–antigen affinity. Indeed, in humanization experiments, affinity is found to be regained after making mutations that are distant from the antigen-binding site and therefore indicative of a structural change, modifying the VH–VL orientation (31–33). In SAbDab, we use the ABangle methodology (15) that characterizes the orientation in an absolute sense using six measures, five angles and a distance. These measures allow for the orientation space of antibodies to be characterized. In SAbDab, we automatically calculate these measures for each *F_V_* region in the database.

## ACCESSING THE DATA

The data in SAbDab can be accessed and filtered in a number of ways. Details of particular structures can be retrieved and viewed or sets of entries can be selected and downloaded. In addition, the entire structural contents of SAbDab can be downloaded.

### Downloads

For each structure, the following files may be downloaded:

1. The pdb structure file
2. A Chothia re-numbered structure file
3. A tab-separated summary file containing information about chain pairings, antigen pairing and other annotations about the structure gathered by SAbDab.

The structure files are available in PDB format. The Chothia re-numbered file contains the coordinates of each atom in the structure. Each antibody residue is renumbered with the Chothia numbering scheme over the variable region of domains. Non-variable region residues are numbered sequentially. Non-antibody chains retain their original residue numbering. The header of each file contains information about the chain types, pairings and antigen pairings. For instance, the structure 1ahw (34) has two heavy–light chain pairs: B–A and E–D. These associate with protein antigen chains C and F, respectively. Thus, the header contains the lines:


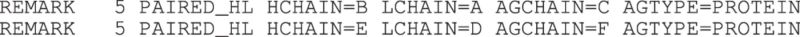


The summary file is a tab-separated.tsv file containing information about chain pairings and details about the structure, for example, experimental details, antigen affinity and species. The first line is the name of each field. Each following line corresponds to a paired heavy and light antibody chain and details corresponding to that pairing. For instance, the first six fields of the summary file for 1ahw appear as:


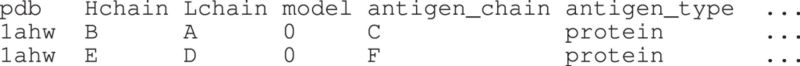


When a user selects any set of structures, they are able to download the files for each structure individually or collectively as a dataset using the ‘download all’ function. In the latter case, a single zip file is created containing an archive of all the selected structures. A single summary file is also created for all the heavy- and light-chain pairings in the selection. This file may also be downloaded separately without the structural data.

### Individual structure information

**Figure 3.**
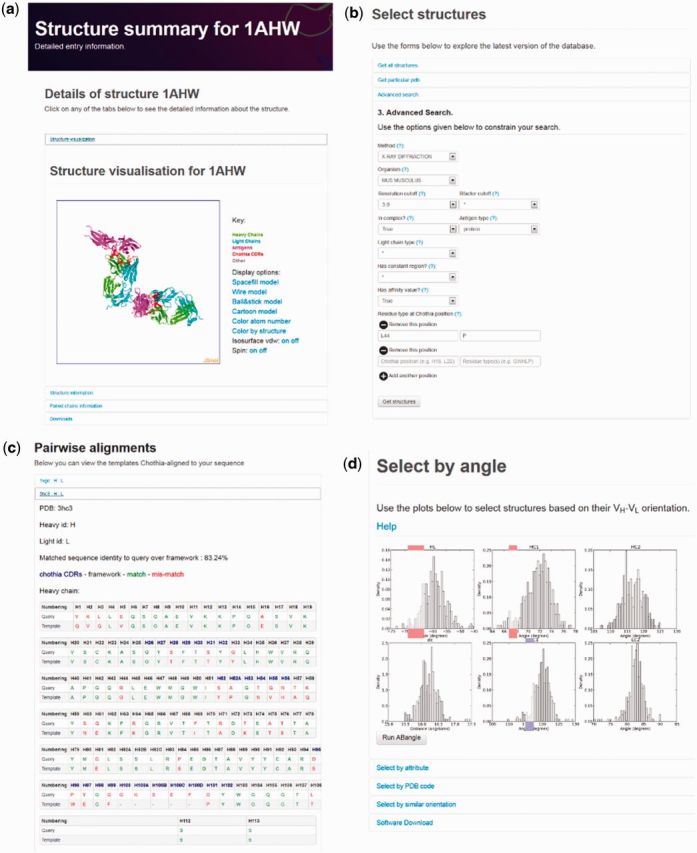
Selected screen-shots of SAbDab (**a**) The structure summary page for an entry in SAbDab. Detailed information about the structure and a visualization of the antibody and antigen is available. (**b**) The advanced search form. Structures may be selected using a number of methods. Here, the advanced search selects the required attributes of each structure in the selection. (**c**) The alignment between a query sequence and a template identified by the template search function. (**d**) The ABangle orientation search tool. Users may select *F_V_* structures by choosing specific regions of the VH–VL orientation space.

An individual structure can be accessed using its PDB accession code (e.g. 1ahw). When a structure is accessed, the user is brought to its summary page as shown in Figure 3a. Here, the structure can be visualized with heavy chain, light chain, antigen and CDRs annotated in different colours. Clicking on the structure information tab shows details including experimental method used to acquire the structure, species information, the number of paired heavy and light chains and, if available, the associated *K_D_* and ΔG values for antibody–antigen binding.

Under the paired chains information tab, further details about each paired heavy and light chain (F*_AB_*) can be found. These include: H and L chain identifiers, the bound state of the F*_AB_*, the IMGT subgroup gene annotations, the Chothia numbered sequence of each chain, information about each CDR and the orientation measures between the VH and VL domains. If present, details of the antigen and its sequence are provided.

The summary page also allows the user the full set of download options. Links are also provided to the original PDB entry and, if available, to the entry for the structure in IMGT.

### Advanced search tool

The advanced search tool (Figure 3b) allows the user to select structures based on a number of attributes. The attributes include experimental method, resolution cut-off (for x-ray structures), r-factor, bound-state (bound or unbound), antigen type, antibody species and antibody light chain type (κ or λ). Users can also specify amino-acid types that must be present at Chothia positions. Similarly, structures can be limited to those that have an associated affinity value or those that have the constant domains of the F*_AB_* region present.

After clicking on the ‘get structures’ button, the user will be presented with a list of structures that satisfy their selection. Basic information is shown for each structure with a link to each entry’s summary page. The ‘downloads’ section of the results page provides options to download the selected structures.

### Non-redundant dataset creation

The antibody and antigen structures in the PDB are highly redundant in terms of sequence. For instance, 6% of the bound antigens in SAbDab are lysozymes. Over representing certain types of antigens in analysis datasets may bias results, especially in the antibody–antigen docking field, where algorithms may be trained using paratope–epitope contacts. To overcome this problem, we provide a non-redundant dataset creation tool. Structures are clustered using cd-hit (35) based on their sequence identity with respect to both antibody and antigen sequences. Users may select sequence identity levels for the antibody and antigen separately and specify other constraints for the structures returned.

### CDR search tools

SAbDab offers a CDR-specific search functionality. A user may select CDRs using similar criteria as in the advanced search tool (‘advanced search’ section). In addition, CDR structures can be searched with respect to their CDR type and length in accordance with different CDR definitions and their membership of structural clusters or canonical classes (‘complimentarity determining regions’ section). SAbDab will return a list of the selected CDR structures. These can be inspected individually or downloaded as described in the ‘downloads’ section. The CDR search tool also allows a non-redundant set of CDR structures to be selected. In this case, only non-identical structures with respect to type, length and sequence are returned. For identical sequences, the structure with the best resolution is returned.

### Template search tool

The template search tool allows users to identify those structures in SAbDab with the highest sequence identity to a given antibody sequence. The returned entries may act as good templates for use in a modelling protocol. Structures can be searched according to their sequence identity over either the heavy or light chain or over both chains at once. Users may specify whether they wish to calculate sequence identity over the full variable region, only the framework regions, only the CDRs or only a particular CDR. An option is also provided that requires each template to have the same structurally equivalent positions as the query sequence i.e. that there are no insertions or deletions between the template and query.

On submission, the top N templates (as specified by the user) are returned, ranked by their matched sequence identity to the query. Each structure may be inspected individually and the Chothia-numbered alignment between the template and the query sequence visualized (Figure 3c). An option is given to download all returned structures individually or *en masse* along with a multiple sequence alignment of the template sequences to the query sequence.

### ABangle search tool

As described in the ‘VH–VL orientation’ section, the orientation between the variable domains can be characterized using six absolute measures. Users can explore the VH–VL orientation space using our ABangle search tool (Figure 3d). The distribution of each measure has been divided into discrete bins. To select structures with a particular orientation, a user may click on one or multiple (or none) bins for each of the distributions. On submission, each F*_V_* region with a VH–VL orientation that falls within the selected orientation range will be returned. Alternatively, the same criteria as in the ‘advanced search’ section can be used to select structures and visualize where they lie in orientation space. For instance, if a user selected structures with a proline (P) at Chothia position L44, these would show a different orientation preference to those with a tryptophan (W) at the same position (15,36,37).

The ‘select by pdb code’ function allows for the selection of a number of individual structures for comparison of their VH–VL orientation. One application of this tool is to compare the VH–VL orientation of antibodies in their bound and unbound form. For example, the HIV-1 neutralizing antibody 50.1 has been crystallized both without and in complex with its peptide antigen (38). These structures have been cited as evidence for conformational changes in the antibody upon antigen-binding. Interestingly, it is the unbound form (1GGC and 1GGB) that has an unusual orientation, while the bound from (1GGI) has an orientation typical of known antibody structures.

## CONCLUSION

SAbDab collects, curates and presents antibody structures from the PDB in an consistent manner. The aim of the database is to provide the antibody research community with a tool to easily create standardized datasets for analysis and to monitor the rapidly increasing amount of available antibody structural data. Automated weekly updates keep the data in SAbDab up to date and ensure the longevity of this resource. The database is complemented by further stand-alone antibody software that can be found under the ‘Tools’ section on the SAbDab front page. We hope that SAbDab provides a useful resource for computational and experimental antibody researchers alike. The database is entirely open-access and available at http://opig.stats.ox.ac.uk/webapps/sabdab.

## FUNDING

Engineering and Physical Sciences Research Council (EPSRC); UCB Pharma; Roche GmbH. Funding for open access charge: EPSRC.

*Conflict of interest statement*. None declared.
